# CGRP Suppresses Protective SiglecF^hi^ Neutrophil Development in Neonatal Group B *Streptococcus* Pneumonia

**DOI:** 10.3390/microorganisms13092119

**Published:** 2025-09-11

**Authors:** Inês Lorga, Ana Sofia Teixeira, Bárbara Carvalho, Joana Soares, Nuno Ribeiro, Marcos S. Cardoso, Joana Cunha, Joana Santos, Regina A. Silva, Manuel Vilanova, Elva Bonifácio Andrade

**Affiliations:** 1 ICBAS—School of Medicine and Biomedical Sciences, Universidade do Porto, 4050-313 Porto, Portugalmanuel.vilanova@icbas.up.pt (M.V.); 2 i3S—Instituto de Investigação e Inovação em Saúde, Universidade do Porto, 4200-135 Porto, Portugal; ana.smmt@gmail.com (A.S.T.);; 3 E2S—Escola Superior de Saúde, Instituto Politécnico do Porto, 4200-072 Porto, Portugal; 4 Área Técnico-Científica Anatomia Patológica, Citológica e Tanatológica, Escola Superior de Saúde, Instituto Politécnico do Porto, 4200-072 Porto, Portugal; 5 Serviço de Anatomia Patológica, Hospital Pedro Hispano, Unidade Local de Saúde de Matosinhos, 4464-513 Matosinhos, Portugal; 6 REQUIMTE/LAQV, Escola Superior de Saúde, Instituto Politécnico do Porto, Rua Dr. António Bernardino de Almeida, 4200-072 Porto, Portugal

**Keywords:** Group B *Streptococcus*, neonatal immunity, innate immunity, pneumonia, lung, neutrophil, SiglecF, CGRP

## Abstract

Neonatal pneumonia, a leading cause of morbidity and mortality, is frequently caused by Group B *Streptococcus* (GBS). The mechanisms underlying protective immunity to this pathogen in the neonatal lung remain incompletely understood. Using a clinically relevant neonatal mouse model of GBS pneumonia, we investigated the immune mechanisms influencing disease severity. We demonstrate that neutrophils are effectively recruited to the lungs of infected neonates, but their phenotype differs with disease severity. In pups with moderate disease, we observe significant infiltration of SiglecF^hi^ neutrophils, a phenotype associated with enhanced phagocytic capacity and bacterial clearance. In contrast, pups with severe disease failed to develop SiglecF^hi^ neutrophils, resulting in reduced bacterial clearance and worsened pathology. We further show that severity is associated with increased expression of calcitonin gene-related peptide (CGRP) in the lungs. CGRP suppressed neutrophil activation into the SiglecF^hi^ phenotype, thereby limiting their antibacterial function. Our findings show that GBS exploits the neuroimmune axis to evade host immunity through CGRP-mediated suppression of neutrophil activation.

## 1. Introduction

Neonatal pneumonia remains a major cause of morbidity and mortality worldwide [1,2]. Among the pathogens responsible for severe neonatal infections, Group B *Streptococcus* (GBS) is the leading cause of invasive diseases, including pneumonia and septicaemia [3]. GBS is a Gram-positive commensal of the genital and gastrointestinal tracts of many healthy adults that can be transmitted vertically from mother to newborn during delivery [3,4,5,6]. The lungs are often the primary organ affected during neonatal GBS infection, given their direct exposure to GBS as a consequence of aspiration of contaminated fluids. In fact, GBS is identified as the most common cause of bacterial pneumonia acquired during delivery, with autopsy data from fatal cases showing extensive lobar or multilobar pneumonia in many infants [7]. However, despite progress in neonatal care, the cellular and molecular mechanisms responsible for pathogen clearance in the lungs remain poorly understood.

In neonates, the immune system differs both phenotypically and functionally from that of adults [8,9]. Neutrophils are key phagocytic cells responsible for bacterial elimination [10]. However, neonatal neutrophils are less efficient than their adult counterparts, exhibiting diminished phagocytic capacity [11], reduced chemotaxis [12], impaired neutrophil extracellular traps (NETs) formation [13], and a generally delayed or weakened response to inflammatory stimuli. These deficiencies contribute to neonates’ increased susceptibility to severe infections [8,9]. Recent work has also shown the heterogeneity of neutrophils populations. Notably, a subset of neutrophils characterised by high expression of SiglecF (sialic acid–binding immunoglobulin-like lectin F), a lectin typically found on mouse eosinophils, has been identified in the lung [14,15,16]. However, the role of SiglecF^hi^ neutrophils in neonatal infections is unknown.

Alongside immune immaturity, increasing evidence points to a critical contribution of the nervous system to immune regulation. Sensory neurons, which densely innervate the lung, can release neuropeptides such as calcitonin gene-related peptide (CGRP) in response to noxious inflammatory stimuli, including bacterial products [17,18,19,20]. CGRP shapes immune responses and has been implicated in pathogen-driven immune evasion across tissues, including the brain, skin, and lungs [6,20,21,22]. In bacterial infections, CGRP has been shown to inhibit neutrophil recruitment, thereby facilitating pathogen persistence [6,21]. Whether similar mechanisms occur during neonatal pneumonia remains unexplored.

Here, we use a neonatal mouse model of vertically transmitted GBS pneumonia to investigate how neuroimmune interactions influence disease severity. We show that while neutrophils are effectively recruited to the neonatal lungs, their activation into a SiglecF^hi^ phenotype is suppressed in severe disease. This inhibition correlates with increased CGRP expression, which limits the antibacterial capacity of neutrophils. Together, our findings uncover a previously unrecognised neuroimmune pathway by which GBS manipulates CGRP signalling to impair neutrophil activation in severe neonatal pneumonia.

## 2. Material and Methods

### 2.1. Ethics Statement

Animal experiments complied with the ARRIVE guidelines, followed the recommendations of the European Convention for the Protection of Vertebrate Animals used for Experimental and Other Scientific Purposes (ETS 123) and Directive 2010/63/EU and Portuguese rules (DL 113/2013). The animal study protocols were approved by the Ethics Committee of Instituto de Investigação e Inovação em Saúde (i3S), and by the competent national authority Direcão Geral de Alimentacão e Veterinária (DGAV) (No. 0421/2022-09-02).

### 2.2. Animal Housing

C57BL/6 mice were bred and maintained at the i3S under specific pathogen-free conditions, under a 12 h light-dark cycle with ad libitum access to food and water. All mice were housed in groups, except for pregnant females, under a controlled environment (22 ± 2 °C; 45–55% relative humidity) in ventilated cages. All efforts were made to minimise animal suffering, including the use of cardboard tunnels and sheets as environmental enrichment. All experiments involving neonates were performed in both male and female, and pregnant females were infected at the age of 8–10 weeks. Investigators were not blinded to group allocation or outcome assessment. No animals were excluded from the analysis.

### 2.3. Bacterial Strains and Culture

GBS strain BM110, a capsular serotype III strain and belonging to the clonal complex 17 (CC17) hypervirulent lineage responsible for more than 80% of meningitis cases [23], or the GBS strain NEM316 belonging to the clonal complex 23 (CC23) [24], were cultured in Todd-Hewitt (TH) broth or agar (Difco Laboratories, Detroit, MI, USA). Overnight cultures of GBS were 1:100 sub-cultured in fresh TH broth and grown at 37 °C until the mid-log phase. Growth was evaluated by monitoring the optical density (OD) at 600 nm (OD_600_ ~0.800) using a spectrophotometer Libra S6+ (Biochrom, Cambridge, UK).

### 2.4. Neonatal Mouse Model of GBS Pneumonia

In the mouse model of infection for human neonatal GBS disease, pregnant C57BL/6 mice were intravaginally (i.vag.) inoculated with 40 μL containing 3–4 × 10^4^ GBS cells at the gestation (G) days 16 and 17, using a micropipette. Pregnant females delivered spontaneously between G19 and G20, and the pups were maintained with their mothers during the experimental period. The day following birth was considered the postnatal day (P) 1. All experiments were controlled with age-matched mice born from PBS sham progenitors. Female mice were randomly assigned to subject groups (infected or control) to eliminate any maternal confounders. Animals born from sham and infected pregnant mice were followed until P5 to monitor their survival curves. The pups’ weight was registered at the indicated time points.

### 2.5. Clinical Evaluation

Infected pups were clinically evaluated daily, as previously described [25], to assess disease progression and predict mortality. Pups were considered severely sick and euthanised if they exhibited any of the following criteria: absence of spontaneous movement, weak suckling (no milk spot), lack of response to tactile stimulation, or a capillary refill time greater than 10 s.

### 2.6. Tissue Collection

Neonatal mice (both male and female), selected at different time points as indicated in the text, were deeply anaesthetised by isoflurane inhalation, transcardially perfused with saline solution, and euthanised by decapitation. The lungs and spleen were aseptically removed, weighed, and handled in agreement with their final use. In the case of RNA analysis, a section of the lung left lobe was immediately snap frozen on dry ice and stored at −80 °C until RNA extraction. When needed, blood was drawn from the mandibular vein using heparin-coated capillaries prior to perfusion. Tissue samples were selected from different litters.

### 2.7. Bacterial Load Analysis

At specific time points (from P1 to P5), the lungs’ superior right lobe was homogenised in 250 μL of PBS with a pellet pestle and a Kimble rotor-stator homogeniser (DWK Life Sciences, Wertheim, Germany). Following homogenisation, serial dilutions were made in sterile saline and plated on TH agar. The plates were incubated overnight at 37 °C, and the number of colony-forming units (CFU) was enumerated.

### 2.8. Histological Analysis

The lung left lobe was fixed in 10% buffered formalin solution (also known as neutral buffered formalin). Subsequently, the tissue was automatically processed for paraffin embedding using the TP10120 tissue processor (Leica^®^, Wetzlar, Germany). After embedding, three µm thick sections were cut from each block using the Histocore Autocut microtome (Leica^®^, Wetzlar, Germany) and stained with either hematoxylin-eosin or Giemsa, following standard protocols. The slides were scanned using the Pannoramic MIDI II automatic digital slide scanner (3DHISTECH, Budapest, Hungary) equipped with a 40× objective. Visualisation and histological analysis of the scanned slides were performed using the CaseViewer software v2.4. The lung parenchyma was divided into four reference anatomical structures, which were used as criteria for analysis: the interstitium (septal, peripheral and axial); airways (terminal and respiratory bronchioles); vessels (arteries, veins and lymphatics); and alveolar spaces (including alveolar ducts). These criteria were used to define representativeness in each histological section. For each pup, three sections per lung were evaluated. Lung histology was rated semi-quantitatively, in a histological score from 0 to 4 based on the percentage of alterations observed (Table 1).

### 2.9. Cell Isolation

Pups (both male and female) were deeply anaesthetised by isoflurane inhalation and euthanised by decapitation. The lungs and spleen were aseptically removed. The lungs were minced into 1–2 mm pieces and digested for 30 min at 37 °C with 1 mg/mL collagenase D (Roche, Basel, Switzerland) in RPMI 1640 medium (HyClone, Cytiva, Marlborough, MA, USA). The reaction was stopped with 2 mM EDTA and 0.5% BSA in PBS (PBE). Following the digestion step, the tissue was gently passed through a 20 gauge (G) needle, followed by a 25G, and gently pressed through 100 μm nylon mesh cell strainer. Suspensions were then centrifuged twice at 70× *g* for 1 min, at 4 °C, and the supernatant collected. Finally, cells were centrifuged at 300× *g* for 4 min at 4 °C, and the supernatant was discarded. Spleens were excised, gently dissociated in a 100 μm cell strainer to yield a single cell suspension.

Red blood cells were lysed in Ammonium-Chloride-Potassium (ACK) solution for 5 min at room temperature and washed in PBE (300× *g* at 4 °C for 4 min). The cell pellets were resuspended in ice-cold FACS buffer [2% FBS (heat-inactivated, Biowest, Bradenton, FL, USA), 2 mM EDTA and 0.01% sodium azide in PBS] or sorting buffer (2% FBS, 1 mM EDTA in PBS) and counted using a Neubauer counting chamber in Trypan blue.

### 2.10. Flow Cytometry

After isolation, cells were washed once with DPBS, and a total of 1 × 10^6^ cells were stained with fixable viability dye (FVD) eFluor 506 or FVD eFluor 780 (eBisocience, Invitrogen, Grand Island, NY, USA) diluted in DPBS for 30 min on ice. Single cell suspensions were sequentially incubated with the following fluorescence-coupled monoclonal antibodies (mAb), used at previously determined optimal dilutions: anti-CD45 FITC (Clone 30-F11), anti-CD69 PE (H1.2F3), anti-CD8 PerCP-Cy5.5 (53–6.7), anti-CD3 Pe-Cy7 (17A2), anti-TCRγδ APC (GL3), anti-TCRβ eFluor 780 (H57-597, eBioscience, San Diego, CA, USA), anti-Siglec-F PE (E50-2440, BD Biosciences, Franklin Lakes, NJ, USA), anti-Ly6C PerCP-Cy5.5 (HK1.4), anti-CD11b Pe-Cy7 (M1/70), anti-CD5 PE (53–7.3, BD Biosciences, Franklin Lakes, NJ, USA), anti-TCRβ PE (H57-597, Invitrogen, Carlsbad, CA, USA), anti-CD19 APC-Cy7 (6D5), anti-IFNγ PE (XMG1.2), anti-TCRγδ Pe-Cy7 (GL3), anti-CD3 APC-Cy7 (17A2), anti-Ly6G AF647 (1A8), anti-Ki67 Alexa Fluor 488 (16A8), anti-TCRVγ4 PE (UC3-10A6), anti-CD45 PerCP-Cy5.5 (30-F11), anti-TCRVγ1 APC (2.11), anti-TCRβ eFluor 450 (H57-597, Invitrogen, Carlsbad, CA, USA), anti-CD44 PerCP-Cy5.5 (IM7), anti-CD4 BV421 (RM4-5), anti-CD62L APC (MEL-14). All antibodies are from Biolegend (San Diego, CA, USA) unless otherwise mentioned. Cells were analysed in FACSCanto II or FACSAria flow cytometers (BD Biosciences, Franklin Lakes, NJ, USA), using FACS Diva software v.8.0.1. Post-acquisition analysis was performed using FlowJo Software v.10 (Tree Star).

### 2.11. Intracellular Staining

For intracellular staining, single-cell suspensions from the lungs were stimulated in complete RPMI medium with PMA (Phorbol 12-Myristate 13-Acetate; 50 ng/mL, Sigma Merck, St. Louis, MI, USA) and Ionomycin (1 μg/mL, Sigma Merck, St. Louis, MI, USA) and Brefeldin A (BFA) (10 μg/mL, Sigma Merck, St. Louis, MI, USA), for 4 h at 37 °C, 5% CO_2_. Cells were washed with ice-cold DPBS (without Ca2+ and Mg2+) and stained for extracellular markers as described above. Cells were then permeabilised with a Foxp3/Transcription Factor Staining Buffer Set (eBioscience, Invitrogen, Grand Island, NY, USA) and stained for 30 min at 4 °C. Then, cells were washed with 100 μL 1× Permeabilisation Buffer and incubated in Permeabilisation Buffer with rat IgG (Sigma Merck, St. Louis, MI, USA) 1:100 and FcBlock 1:500 for 10 min, at 4 °C. Intracellular staining mix, containing anti- IFN-γ-PE (clone XMG1.2, Biolegend, San Diego, CA, USA) and anti- IL-17-APC (clone TC11-18H10.1, Biolegend, San Diego, CA, USA) was added and the cells were incubated for 1 h, at room temperature. Cells were washed first in Permeabilisation buffer and then in FACS buffer and resuspended in FACS buffer.

### 2.12. RNA Extraction

Tissues were removed and snap-frozen using liquid nitrogen. Thawed tissues were immediately homogenised in 1 mL of TRIzol reagent (Invitrogen, Grand Island, NY, USA). Chloroform (200 μL) was added, samples were mixed, and centrifuged (12,000× *g*, 15 min, 4 °C). The upper phase was collected, and RNA was extracted using the Ambion Pure linkTM RNA Mini Kit (Thermo Fisher Scientific, Waltham, MA, USA). RNA quality was evaluated using Experion^TM^ Eukaryote Total RNA StdSens and RNA concentration was assessed Nanodrop 1000 (Thermo Scientific, Waltham, MA, USA).

### 2.13. Real-Time Quantitative PCR (RT-qPCR)

RNA was reverse-transcribed into cDNA using the SuperScriptTM IV First-Strand Synthesis System (Invitrogen, Grand Island, NY, USA), following the manufacturer’s guidelines. RT-qPCR reactions were performed on a CFX384 Real-Time PCR Detection System (Bio-Rad, Hercules, CA, USA) lightcycler using iTaq Universal SYBR Green Supermix (Bio-Rad, Hercules, CA, USA). cDNA was diluted at 1:5 prior reaction and each sample was analysed in duplicate. Relative gene expression levels were normalised to *Hprt* mRNA levels in each sample using the 2^−ΔCt^ method. The primer sequences used for the RT-qPCR of the target gene can be found in Table 2.

### 2.14. Bacterial Killing Assays

For bacterial killing assays, mouse neutrophils were FACS sorted from the lungs of P5 pups, based on the expression of SiglecF (CD45^+^Ly6G^+^CD11b^+^SiglecF^hi^ and CD45^+^Ly6G^+^CD11b^+^SiglecF^neg/low^). Neutrophils were placed in coculture with GBS BM110 at a multiplicity of infection (MOI) of 5, in 200 μL of RPMI complete medium (without antibiotics). Co-cultures were centrifuged at 300× *g* for 5 min and incubated for 1 h at 37 °C in 5% CO_2_. Upon incubation, cells were washed and further incubated with 200 μg/mL gentamicin for 30 min, at 37 °C in 5% CO_2_. Cells were washed twice in PBS and lysed by resuspension in 100 μL of ice-cold ddH_2_O. The number of intracellular bacteria was determined by serially dilution and plating in TH agar.

### 2.15. Neutrophil Stimulation with CGRP In Vitro

Following euthanasia, femurs and tibias were dissected from mice. Neutrophils were isolated from the bone marrow as described [26]. Briefly, bone marrow cells were flushed out using 1× HBSS 0.38% sodium citrate buffer in a 5 mL syringe and a 25G needle. Cells were then gently disaggregated by repeatedly aspirating and expressing the solution using an 18G needle and a 5 mL syringe to yield a single-cell solution and centrifuged for 6 min at 230× *g* at room temperature. Cells were washed with 1× HBSS 0.38% sodium citrate buffer and the pellet resuspended in 2 mL 1× HBSS 0.38% sodium citrate buffer. Percoll working solution (90%) was prepared with 10× HBSS buffer (without Ca2^+^ and Mg^2+^ or phenol red) and further diluted with 1× HBSS 0.38% sodium citrate to prepare the 72%, 64% and 52% Percoll (Cytiva, Marlborough, MA, USA) dilutions. Slowly, the cell suspension was added on top of the Percoll gradient and centrifuged at 1545× *g*, 30 min, at room temperature. The enriched population of neutrophils was collected at the 72–64% interphase. After isolation, cells were washed, counted and the purity assessed by flow cytometry. Neutrophils were incubated for 4 h at 37 °C with heat-killed GBS BM110 at a MOI of 10 and 50, without or with CGRP (100 nM, Peptide Institute. Inc., Osaka, Japan). After, cells were washed, surface stained with appropriate markers, fixed with PFA and analysed by flow cytometry.

### 2.16. Data Analysis and Statistics

Data were plotted and analysed with the GraphPad Prism software (v.10.1.1, GraphPad Software LLC, San Diego, CA, USA). Means and standard error of the means (SEM) were calculated, corresponding to the indicated independent experiments. The sample size was calculated using G*Power software (version 3.1.9.6 for Mac OS). The number of biological replicates (*n*) and the number of independent experiments are indicated in the figure legends. No data were excluded. The log-rank (Mantel–Cox) test was used to analyse the survival curve. Student’s *t*-test was used when analysing two different groups. Statistical analysis of changes in two or more groups compared at multiple time points was analysed by One- or Two-way ANOVA. Normality was verified by the Shapiro–Wilk normality test. CFU data were log10 transformed. Differences were considered significant for *p* ≤ 0.05. Sample sizes are expressed in each figure legend.

## 3. Results

### 3.1. Vertical Transmission of GBS Leads to Lung Pathology

**Figure 1:**
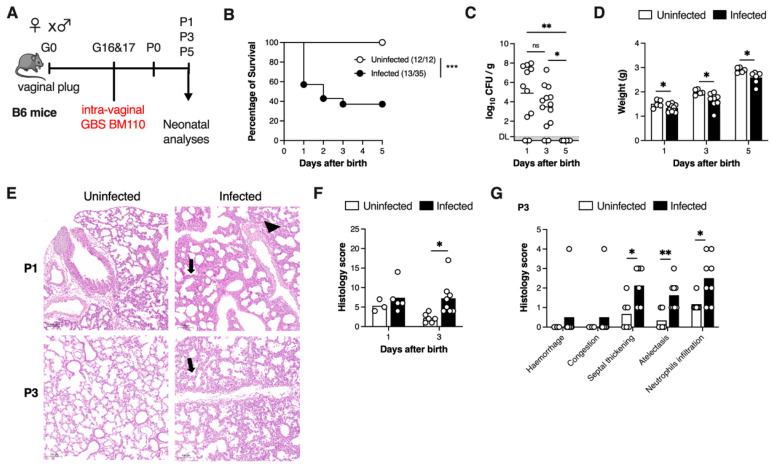
GBS vertical transmission leads to lethal neonatal pneumonia. (**A**) Schematic illustration of the colonisation model. Pregnant C57BL/6 female mice were intravaginally inoculated with 4 × 10^4^ CFU of GBS hypervirulent strain BM110, at gestational days 16 and 17. (**B**) Kaplan–Meier survival curve of neonatal mice born from GBS-colonised dams, monitored over 5 days. The numbers in parentheses represent the number of animals that survived versus the total number of animals born. (**C**) Newborn mice were sacrificed at postnatal days 1, 3, and 5 and the lung bacterial loads were determined. Data are presented as mean ± SEM [*n* = 11 (P1); *n* = 13 (P3); *n* = 6 (P5)]. Each symbol indicates data from a single pup. Comparisons by One-way ANOVA. (**D**) Newborn mice were weighted at postnatal days 1, 3 and 5. Data presented as mean ± SEM [*n* = 5 (Uninfected); *n* = 7–11 (Infected)]. Each symbol indicates data from a single pup. Comparisons by Student’s *t*-test. (**E**) Representative images of H&E-stained lung tissue slices of uninfected and infected mice at the indicated timepoints. Scale bar 100 μm. Black arrows and arrowhead indicate thickening of the interalveolar septum (septal thickening) and haemorrhage, respectively. (**F**) Global histopathology score was attributed to lungs stained with H&E. Each symbol indicates data from a single pup. (**G**) Histological analysis of lungs of Uninfected (*n* = 6) and GBS-infected (*n* = 8) pups at P3. Each symbol indicates data from a single pup. (**F**,**G**) Data shown as mean ± SEM. Comparisons by Student’s *t*-test. Results are pooled from two to four independent experiments. Statistical differences (*p* values) between groups are indicated. * *p* < 0.05, ** *p* < 0.01, *** *p* < 0.001. ns, not significant.

To investigate whether vertical transmission of GBS would constitute a suitable model for studying neonatal pneumonia, we first adapted our previously described model of GBS mother-to-progeny transmission [27] to the C57BL/6 mouse model (Figure 1A). Survival curve analysis demonstrated that approximately 60% of pups born from dams colonised with GBS BM110 succumbed to disease within the first three days of life, with the highest mortality (~40%) occurring between P0 and P1 (Figure 1B), likely due to fulminant septicaemia. This outcome contrasted significantly with the complete absence of mortality in the control group. No more deaths were registered after day 3 (Figure 1B). To assess the progression of infection, bacterial load was quantified in the neonatal lungs at different time points after delivery. The highest bacterial burden was observed at P1, with a slight reduction at P3, albeit not statistically significant. By P5, bacteria were no longer detected (Figure 1C). Consistent with bacterial load, infected pups showed significantly reduced body weight gain compared to uninfected controls (Figure 1D).

GBS early-onset disease, occurring in the first week of life, is often accompanied by respiratory complications, including hypoxia and pulmonary hypertension, leading to pneumonia [5,7,28], a condition known to cause tissue pathology [29]. Thus, pulmonary disease severity was assessed by performing H&E and Giemsa staining of lung sections at time points when bacterial burden was detectable (Figure 1E and Supplementary Figure S1). Four anatomic structures were defined when observing the lung sections, namely the interstitium, airways, alveoli, and blood vessels. Interestingly, both infected and uninfected pups displayed mild histopathological alterations at P1 (Uninfected, 7.33 ± 0.88; Infected, 9.00 ± 1.817) (Figure 1F). This likely reflects physiological postnatal lung adaptation rather than infection-driven pathology. However, by P3, infected animals exhibited a significant increase in the total histological score compared to their uninfected counterparts (Figure 1F). When analysing each of these findings individually, we found that infected animals were characterised by increased septal thickening, atelectasis, higher neutrophil infiltration, and the presence of alveolar macrophages (Figure 1G). These findings suggest that infection is the factor leading to a worse condition of the neonatal lungs, as reflected by a higher histopathology score.

Collectively, these data indicate that vertical transmission of GBS results in lungs infection and pulmonary lesions consistent with pneumonia in neonates.

### 3.2. GBS Neonatal Pneumonia Does Not Impair Neutrophil Recruitment to the Lungs

**Figure 2:**
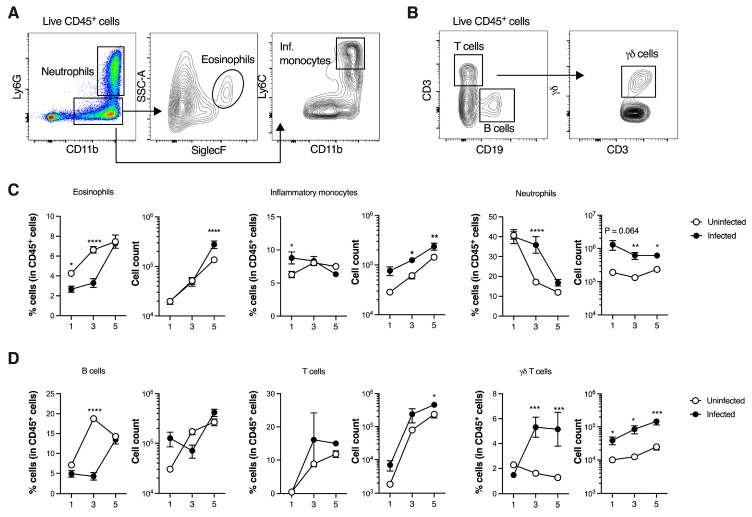
Immune cellular dynamics during GBS neonatal pneumonia. Pregnant C57BL/6 female mice were intravaginally inoculated with 4 × 10^4^ CFU of GBS hypervirulent strain BM110, at gestational days 16 and 17. Animals from uninfected and infected groups were sacrificed at indicated time points and the lung was collected for flow cytometry analysis. (**A**) Representative gating strategy used to define myeloid cells, gated within live CD45^+^ cells. Neutrophils were defined as CD11b^+^Ly6G^+^, eosinophils were defined as CD11b^+^Ly6G^-^SiglecF^+^SSC-A^int/hi^, inflammatory monocytes were defined as CD11b^+^Ly6G^-^Ly6C^hi^. (**B**) Representative gating strategy used to define lymphoid cells, gated within live CD45^+^ cells. T cells were defined as CD3^+^CD19^-^, B cells were defined as CD3^-^CD19^+^, γδ T cells were defined as CD3^+^CD19^-^γδ^+^. (**C**) Frequency and number of indicated myeloid cells. Data are presented as mean ± SEM. Each symbol represents data from a single pup [*n* = 6 (P1, uninfected); *n* = 11 (P1, infected); *n* = 11 (P3, uninfected); *n* = 10 (P3, infected); *n* = 7–8 (P5, uninfected); *n* = 8 (P5, infected)]. Comparisons by two-way ANOVA. (**D**) Frequency and number of indicated lymphoid cells. Data is presented as mean ± SEM. Each symbol represents data from a single pup [*n* = 6 (P1, uninfected); *n* = 11 (P1, infected); *n* = 5–8 (P3, uninfected); *n* = 8–10 (P3, infected); *n* = 8 (P5, uninfected); *n* = 8 (P5, infected). Comparisons by two-way ANOVA. Results are pooled from two to four independent experiments. Statistical differences (*p* values) between groups are indicated. * *p* < 0.05, ** *p* < 0.01, *** *p* < 0.001, **** *p* < 0.001.

The immune system plays a crucial role in protecting against bacterial pneumonia in adults [29,30]. However, the mechanisms contributing to protective or detrimental immunity and subsequent pathology in neonatal lungs remain poorly understood. To address this, the main myeloid and lymphoid cell populations in the lungs of infected pups were analysed during the first five days of life and compared to age-matched uninfected controls using flow cytometry (Figure 2A,B, gating strategy). A significant decrease in the frequency of eosinophils was found at P1 and P3 in the lungs of infected pups compared to uninfected controls (Figure 2C). However, the total number of eosinophils did not differ between groups. By P5, although their relative percentage was similar between groups, the total numbers were significantly increased in infected animals. The total numbers of inflammatory monocytes were also increased in the lungs of infected pups compared to uninfected controls, reaching significance at P3 and P5 (Figure 2C). Strikingly, neutrophils exhibited the most pronounced increase among the myeloid populations, with a significant increase in both frequency and cell number in infected pups, at P3.

In the lymphoid compartment, γδ T cells were markedly altered in infected neonates. Both their frequency and numbers were significantly increased at P3 and P5, suggesting that they could be recruited or expanding in response to infection (Figure 2D). As for the total CD3^+^ T cells, this population only showed a significant increase in cell counts at P5, with higher numbers in infected pups compared to controls. In control pups, the frequency of B cells quickly increased from P1 to P3. However, this was not observed in infected animals, where the percentage of B cells was significantly reduced at P3. By P5, no differences were observed in the frequency of B cells between groups (Figure 2D).

**Figure 3:**
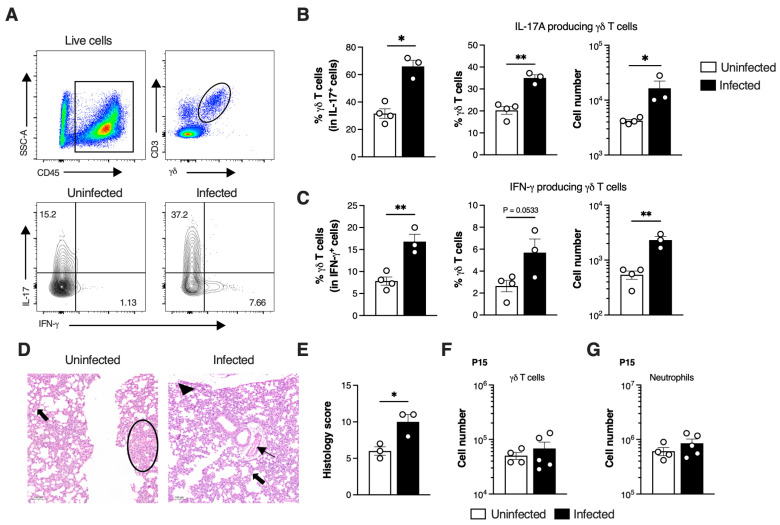
γδ T cells are a major source of IL-17A during GBS neonatal pneumonia. Pregnant C57BL/6 female mice were intravaginally inoculated with 4 × 10^4^ CFU of GBS hypervirulent strain BM110, at gestational days 16 and 17. Animals from uninfected and infected groups were sacrificed at P3 or P15. (**A**) Representative gating strategy used to define γδ T cells and IL-17A and IFN-γ positive cells. Live cells were gated in SSC-A vs. CD45^+^ cells, and γδ T cells were defined as CD3^+^γδ^+^. (**B**,**C**) Frequency and number of lung IL-17- (**B**) and IFN-γ- (**C**) producing γδ T cells at P3. Data is presented as mean ± SEM. Each symbol indicates data from a single pup [*n* = 4 (uninfected); *n* = 3 (infected)]. Comparisons by Student’s *t*-test. (**D**) Representative images of H&E-stained lung tissue slices of uninfected and infected mice at P15. Scale bar 100 μm. Thickening of the interalveolar septum, haemorrhage, vascular congestion, and atelectasis were, respectively, indicated by black arrows, arrowhead, thin black arrow, and ellipse. (**E**) Global histopathology score was attributed to lungs stained with H&E. Data are shown as mean ± SEM. Each symbol represents data from a single pup (*n* = 3). Comparisons by Student’s *t*-test. (**F**) Number of γδ T cells in the lungs at P15. Data is presented as mean ± SEM [*n* = 4 (uninfected); *n* = 5 (infected)]. Comparisons by Student’s *t*-test. (**G**) Number of neutrophils in the lungs at P15. Data is presented as mean ± SEM [*n* = 4 (uninfected); *n* = 5 (infected)]. Comparisons by Student’s *t*-test. Results are representative of two independent experiments. Statistical differences (*p* values) between groups are indicated. * *p* < 0.05; ** *p* < 0.01.

Neutrophil recruitment to infected tissues is a hallmark of inflammation and bacterial infections and is partially driven by IL-17A (referred hereon as IL-17). Thus, we sought to determine whether this cytokine was increased during neonatal pneumonia and its cellular source. γδ T cells are an important source of IL-17, available to respond in neonates, and we have found that these cells were increased during neonatal GBS pneumonia. When analysing their phenotype, our data revealed that γδ T cells were predominantly IL-17 producers in infected neonatal lungs, after ex vivo stimulation with PMA and ionomycin, although IFN-γ-producing γδ T cells were also detected at P3 (Figure 3A–C). While infection led to increased frequencies of both IL-17- and IFN-γ-producing γδ T cells, this increase was more pronounced in the IL-17-producing subset (Figure 3A–C). Upon gating on IL-17-positive cells, we found that the lungs of GBS-infected pups presented high frequencies of γδ T cells among total IL-17-producing cells, accounting for approximately 70% of total IL-17-producing cells in this tissue (Figure 3B). Given that γδ T cells are associated with lung homeostasis, tissue repair and maintenance of barrier integrity [31,32], we investigated whether γδ T cells remained altered at later infection time points. Since two weeks after birth entails the peak of pulmonary immune system development during the alveolarisation period and the beginning of microvascular maturation [33,34], we ascertained whether lung tissue presented pathological alterations. Infected animals presented a larger area of tissue with histopathological abnormalities, with at least three distinct pathological findings present simultaneously, suggesting that survivors might develop respiratory sequelae (Figure 3D,E). At this time point, no differences were observed in the number of γδ T cells or neutrophils between infected and uninfected animals (Figure 3F,G). Additionally, evaluation of other myeloid cell numbers (eosinophils and inflammatory monocytes) revealed no differences between groups, either in the lungs or in the spleen (Supplementary Figure S2A,B). To further explore the phenotype of γδ T cells, their residency profile was analysed using the residency markers CD69, CD44 and CD62L (Supplementary Figure S2C, gating strategy). Our data revealed that γδ T cells in the lungs of animals surviving neonatal pneumonia exhibited an activated CD69^+^CD44^+^CD62L^-^ profile, consistent with tissue-resident effector T cells (Supplementary Figure S2C–E).

Altogether, these findings show that infected pups are able to efficiently recruit neutrophils into the lungs early in the course of infection. Furthermore, γδ T cells mount a rapid response to GBS infection and are a major source of IL-17, indicating their potential role in neutrophil recruitment.

### 3.3. SiglecF^hi^ Neutrophils Accumulate in the Neonatal Lungs During Moderate Disease

**Figure 4:**
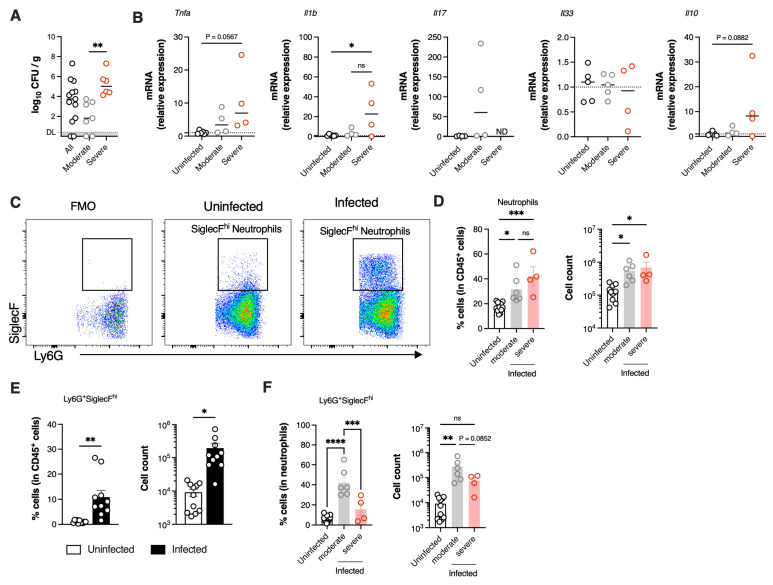
SiglecF^hi^ neutrophils accumulate in the lungs during moderate neonatal pneumonia. Pregnant C57BL/6 female mice were intravaginally inoculated with 4 × 10^4^ CFU of GBS hyper-virulent strain BM110, at gestational days 16 and 17. Animals from uninfected and infected groups were sacrificed at P3. Animals’ stratification was made by dividing data into All (all infected animals), Moderate (infected animals with moderate signs of disease) and Severe (infected animals with severe signs of disease). (**A**) Bacterial loads in the lungs determined by disease severity. Data are presented as mean [*n* = 13 (all); *n* = 7 (moderate); n = 6 (severe)]. Each symbol indicates data from a single pup. Comparisons by One-way ANOVA. (**B**) Relative gene expression of the indicated pro- and anti-inflammatory cytokines evaluated by RT-qPCR, normalised for the reference gene *Hprt*. Samples are stratified by disease severity. Data are presented as mean ± SEM [*n* = 5 (uninfected); *n* = 4–5 (moderate); *n* = 4 (severe)]. Each symbol indicates data from a single pup. Comparisons by One-way ANOVA. (**C**) Representative gating strategy used to define neutrophils’ profile. Cells were gated within live CD45^+^ cells. Neutrophils were defined as CD11b^+^Ly6G^+^. Profile of neutrophils was defined as CD11b^+^Ly6G^+^SiglecF^hi^ or CD11b^+^Ly6G^+^SiglecF^neg/low^. FMO, Fluorescence minus one. (**D**) Frequency and number of neutrophils in the lungs of uninfected, moderate and severe groups. Data is presented as mean ± SEM [*n* = 11 (uninfected); *n* = 6 (moderate); *n* = 4 (severe)]. Each symbol indicates data from a single pup. Comparisons by One-way ANOVA. (**E**) Frequency and number of SiglecF^hi^ neutrophils in uninfected and infected animals. Data are presented as mean ± SEM [*n* = 11 (uninfected); *n* = 10 (infected)]. Each symbol indicates data from a single pup. Comparisons by Student’s *t*-test. (**F**) Frequency and number of SiglecF^hi^ neutrophils in the lungs of infected pups according to disease severity. Data are presented as mean ± SEM [*n* = 11 (uninfected); *n* = 6 (moderate); *n* = 4–5 (severe)]. Each symbol indicates data from a single pup. Comparisons by One-way ANOVA. Results are pooled from two to four independent experiments. Statistical differences (*p* values) between groups are indicated. * *p* < 0.05; ** *p* < 0.01; *** *p* < 0.001, and **** *p* < 0.0001. ND, not detected. ns, not significant. DL, detection level.

Given that infected pups efficiently recruited neutrophils to infected tissue, we sought to understand the underlying factors contributing to the observed mortality. Our analysis was stratified by disease severity at P3, a time point at which physical distress is clearly manifested in severely affected animals. When bacterial colonisation in the lungs was stratified by severity (moderate or severe), we found that animals with severe disease had a significantly higher bacterial load compared to those with moderate disease, indicating a diminished capacity to control the infection (Figure 4A). To evaluate whether disease severity was associated with increased local inflammation, the expression of genes encoding key pro-inflammatory cytokines, such as TNF-α, IL-1β, IL-33, IL-17, and the anti-inflammatory cytokine IL-10 was determined in the lungs of pups presenting moderate or severe pathology by real-time qPCR (RT-qPCR). The results revealed a relatively weak pro-inflammatory profile, with a significant increase only in *Il1b* expression in animals with severe disease, compared to uninfected controls (Figure 4B). Despite differences in bacterial load, the expression of *Tnfa*, *Il33*, *Il17* and *Il10* did not significantly differ between moderate and severe disease groups. However, a tendency towards higher *Tnfa* expression was observed in pups with severe disease (*p* = 0.0567).

As efficient neutrophil recruitment into the lungs was observed early upon infection (Figure 2C), we next assessed whether the neutrophil population was altered in pups with severe disease. Both pups with moderate and severe disease had significantly higher frequency and numbers of neutrophils in the lungs as compared to uninfected controls (Figure 4C,D). Interestingly, no differences in this population were observed between pups with moderate and severe GBS disease (Figure 4D). Given that neutrophils are a heterogeneous population, we hypothesised that the ones present in the lungs during neonatal pneumonia could exhibit distinct phenotypes depending on lung pathology. We identified a subset of neutrophils with a SiglecF^hi^ phenotype (Figure 4C) that was present at higher numbers in the lungs of infected animals, when compared to uninfected controls, where this population was rare (Figure 4E). Alveolar macrophages (AM) colonise the lung shortly after birth and express high levels of SiglecF [35]. CD11b^+^Ly6G^+^SiglecF^hi^ neutrophils lacked the expression of F4/80 and CD64, characteristic markers of AM (Supplementary Figure S3A). Further exclusion of AM and eosinophils from the analyses confirms their identity as neutrophils (Supplementary Figure S3B). The results show that with moderate disease, there is a significant increase in the frequency of SiglecF^hi^ neutrophils within the total neutrophil population. In contrast, this increase was not pronounced in animals with severe disease, suggesting that the presence of SiglecF^hi^ neutrophils could be important for bacterial clearance and contribute to neonatal survival (Figure 4F).

### 3.4. SiglecF^hi^ Neutrophils Exhibit Enhanced Phagocytic Capacity and Contribute to Bacterial Clearance

**Figure 5:**
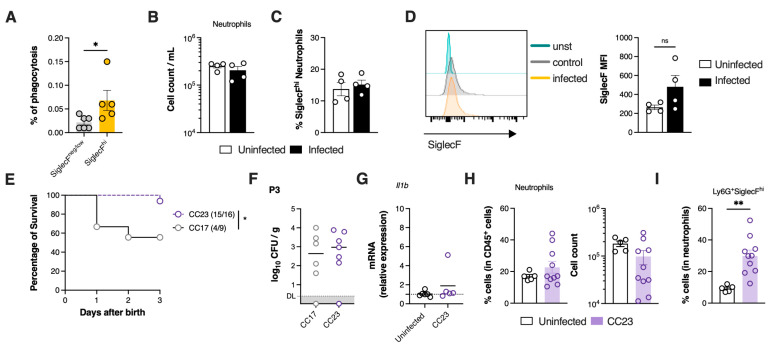
SiglecF^hi^ neutrophils have higher phagocytic capacity and contribute to host defence in GBS neonatal pneumonia. (**A**) Gentamycin protection assay for phagocytosis analysis of SiglecF^hi^ and SiglecF^neg/low^ sorted from the neonatal mouse lung after 1 h incubation with GBS at a MOI of 5 and enumeration of CFUs determined by serial dilution plating. Pooled data from two independent experiments (*n* = 6, SiglecF^neg/low^; *n* = 5, SiglecF^hi^). Each symbol indicates a replica. Comparisons by Student’s *t*-test. (**B**–**F**) Pregnant C57BL/6 female mice were intravaginally inoculated with 4 × 10^4^ CFU of GBS hyper-virulent strain BM110, at gestational days 16 and 17. Animals from uninfected and infected groups were sacrificed at P3. (**B**) Cell number of circulating neutrophils per mL of blood. Data are presented as mean ± SEM pooled (*n* = 4 per group). Each symbol indicates data from a single pup. Comparisons by Student’s *t*-test. (**C**) Percentage of SiglecF^hi^ neutrophils in circulation. Data are presented as mean ± SEM (*n* = 4 per group). Each symbol indicates data from a single pup. Comparisons by Student’s *t*-test. (**D**) Representative histograms and mean fluorescence intensity (MFI) due to neutrophil surface SiglecF staining. Yellow line, infected pups with moderate disease; Grey line, uninfected pups; Green line, unstained. Data are presented as mean ± SEM (*n* = 4 per group). Each symbol indicates data from a single pup. Comparisons by Student’s *t*-test. (**E**–**I**) Pregnant C57BL/6 female mice were intravaginally inoculated with 4 × 10^4^ CFU of GBS hypervirulent strain BM110 (CC17) or the NEM316 strain (CC23), at gestational days 16 and 17. (**E**) Kaplan–Meier survival curve of neonatal mice born from GBS-colonised dams, monitored over 3 days. The numbers in parentheses represent the number of animals that survived versus the total number of animals born. (**F**) Newborn mice were sacrificed at postnatal day 3 and the lung bacterial loads were determined. Data are presented as mean ± SEM [*n* = 6 (CC17); *n* = 7 (CC23)]. Comparisons by Student’s *t*-test. (**G**) Relative expression of *Il1b* analysed by RT-qPCR and normalised for the reference gene *Hprt*. Data are presented as mean ± SEM [*n* = 6 (uninfected); *n* = *5* (CC23)]. Comparisons by Student’s *t*-test. (**H**) Frequency and number of neutrophils in the lungs of pups born from CC23 colonised dams. Data are presented as mean ± SEM [*n* = 5 (uninfected); *n* = 10 (CC23)]. Comparisons by Student’s *t*-test. (**I**) Frequency of Ly6G^+^SiglecF^hi^ neutrophils in the lungs. Data are presented as mean ± SEM [*n* = 5 (uninfected); *n* = 10 (CC23)]. Comparisons by Student’s *t*-test. Each symbol indicates data from a single pup. Results are pooled from two to four independent experiments. Statistical differences (*p* values) between groups are indicated. * *p* < 0.05, ** *p* < 0.01. ns, not significant.

Having found that neutrophils are efficiently recruited into the lungs of neonatal mice during GBS pneumonia and that their phenotype varies with disease severity, we sought to investigate whether SiglecF^hi^ neutrophils play a role in local bacterial clearance. To assess their potential contribution, the phagocytic capacity of SiglecF^hi^ neutrophils was evaluated by performing an ex vivo bacterial killing assay. Neutrophils were sorted from the neonatal lungs based on their expression of SiglecF (CD45^+^Ly6G^+^CD11b^+^SiglecF^hi^ and CD45^+^Ly6G^+^CD11b^+^SiglecF^neg/low^) and incubated with GBS (MOI = 5). A significant increase in phagocytic capacity of the SiglecF^hi^ subset compared to the SiglecF^neg/low^ neutrophils (Figure 5A). To further elucidate the origin of SiglecF^hi^ neutrophils during GBS pneumonia, the presence of this population was assessed in the peripheral blood and spleen of infected pups at P3, comparing the results with uninfected controls. The total number of circulating neutrophils, as well as the frequency of SiglecF^hi^ subset among neutrophils, did not differ between the groups (Figure 5B,C). Additionally, analysis of mean fluorescence intensity (MFI) due to cell surface SiglecF staining revealed no significant differences (Figure 5D). Similarly, no differences in the frequency and number of neutrophils were observed in the spleen (Supplementary Figure S4A), nor in the SiglecF^hi^ subset (Supplementary Figure S4B), suggesting that SiglecF^neg/low^ neutrophils may acquire the SiglecF^hi^ phenotype locally in response to stimuli received within the lung environment.

To determine whether the differences in neutrophil phenotypes were exclusively induced by the hypervirulent CC17 GBS strain used, and to model the disease in a less severe scenario, we conducted infection studies using a non-CC17 GBS strain (NEM316), which belongs to capsular serotype III and CC23 [36]. Pups born from CC23-colonised progenitors showed significantly increased survival than pups born from CC17-colonised mothers (Figure 5E). Moreover, we found no significant difference in bacterial burden between CC23 and CC17 infected pups presenting moderate disease (Figure 5F). Concomitantly, the expression of *Il1b* in CC23-infected pups did not differ significantly from uninfected controls (Figure 5G). Notably, no significant differences in either the percentage or number of neutrophils were detected between uninfected and CC23-infected pups (Figure 5H). However, approximately 30% of neutrophils in the lungs of CC23-infected pups exhibited the SiglecF^hi^ phenotype, similarly to the results obtained from animals infected with the virulent strain and exhibiting moderate disease (Figure 5I).

In summary, the accumulation of SiglecF^hi^ neutrophils in the lungs of the infected animals, combined with their increased phagocytic capacity, sustains the hypothesis that this immune population plays a critical role in bacterial clearance during neonatal GBS pneumonia.

### 3.5. Severe GBS Pneumonia Induces CGRP Expression in the Lungs Dampening SiglecF^hi^ Neutrophil Development

**Figure 6:**
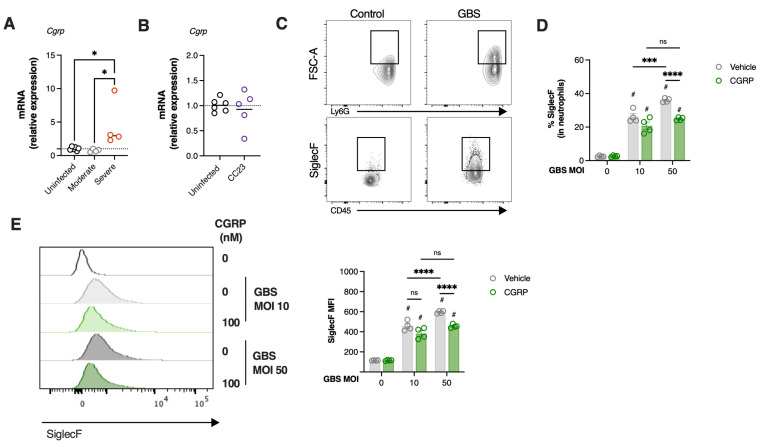
CGRP suppress neutrophils activation during GBS infection. (**A**,**B**) Pregnant C57BL/6 female mice were intravaginally inoculated with 4 × 10^4^ CFU of GBS BM110 (CC17) or NEM316 (CC23), at gestational days 16 and 17. Animals from uninfected and infected groups were sacrificed at P3. For pups born from CC17-colonised progenitors, samples are stratified by disease severity as Moderate (infected animals with moderate signs of disease) and Severe (infected animals with severe signs of disease). Relative gene expression of *Cgrp* was evaluated by RT-qPCR, normalised for the reference gene *Hprt*. Data are presented as mean ± SEM pooled from 4 independent experiments [*n* = 6 (uninfected); *n* = 4 (moderate); *n* = 4 (severe); *n* = 5 (CC23)]. Each symbol indicates data from a single pup. Comparisons by One-way ANOVA or Student’s *t*-test. (**C**–**E**) Mouse neutrophils were incubated with heat-killed GBS at the indicated MOI in the presence of CGRP or vehicle for 4 h. The frequency of SiglecF^hi^ neutrophils was determined by flow cytometry analyses. © Representative gating strategy used to analyse neutrophils. (**D**) Frequency of SiglecF^hi^ among neutrophils in the indicated groups. Data is presented as mean ± SEM. (**E**) Left, Histogram overlays of representative examples of SiglecF channel fluorescence of neutrophils incubated with the indicated conditions. Right, quantification of SiglecF on neutrophils, presented as mean fluorescence intensity (MFI). Data are presented as mean ± SEM pooled from 2 independent experiments (*n* = 4 samples/group). Comparisons by two-way ANOVA. In panels D and E, statistical differences (*p* values) between groups are indicated, # symbols on the top of bars represent a comparison with controls. * *p* < 0.05, *** *p* < 0.001, **** *p* < 0.0001. ns, not significant.

Nociceptor activation triggers the release of calcitonin gene-related peptide (CGRP) from nerve terminals [20] and has been implicated in bacteria-induced neuroimmune evasion by inhibiting neutrophil recruitment and killing [6,21]. Thus, we assessed in our vertical transmission model of GBS pneumonia, the relative expression of *Cgrp* in the lungs of infected neonates. A significant increase in *Cgrp* expression was observed in animals with severe disease compared to both uninfected controls and animals with moderate disease (Figure 6A). In contrast, pups infected with the CC23 strain did not present altered *Cgrp* gene expression (Figure 6B).

Given that at this time point, animals with severe disease were still capable of efficiently recruiting neutrophils while also upregulating the *Cgrp* gene, we next examined whether CGRP could suppress the conversion of neutrophils into the SiglecF^hi^ phenotype. To this end, neutrophils isolated from the bone marrow were incubated with different MOI of heat-killed GBS in the presence of CGRP or vehicle control. Bacterial stimulation resulted in increased neutrophil size, as indicated by higher neutrophil forward scatter (FSC), suggesting increased phagocytosis (Figure 6C). Moreover, GBS stimulation significantly increased the proportion of cells displaying the SiglecF^hi^ phenotype in a dose-dependent manner (Figure 6D). However, when neutrophils were incubated with CGRP, this increase in the SiglecF^hi^ population was significantly inhibited, particularly at the higher dose (MOI = 50) (Figure 6D). In line with this finding, CGRP also led to a reduced increase in SiglecF MFI (Figure 6E).

**Figure 7:**
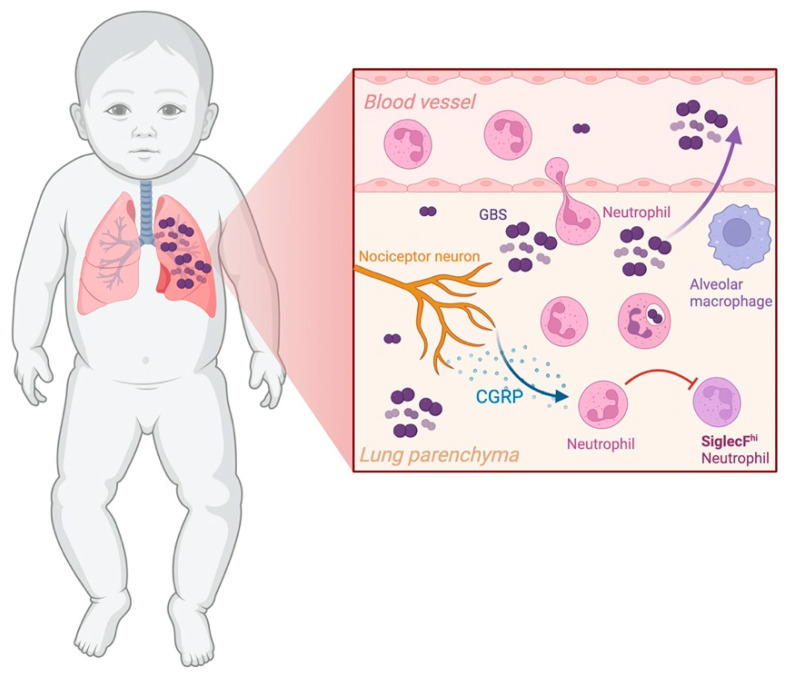
Schematic representation of a proposed mechanism by which Group B *Streptococcus* (GBS) hijacks the neuro-immune axis to evade the host innate immune system. GBS interact and activate nociceptor neurons in the lungs, which releases the neuropeptide CGRP. CGRP appears to hinder neutrophil activation into SiglecF^hi^ neutrophil, a profile associated with increased bacterial clearance. This illustration is for explanatory purposes and is not drawn to scale. Created in BioRender. Andrade, E. (2025) https://BioRender.com/i27v945 (3 September 2025).

In conclusion, our findings suggest that CGRP upregulation during severe GBS pneumonia impairs the development of SiglecF^hi^ neutrophils, potentially hindering effective bacterial clearance and contributing to worsened disease outcomes (Figure 7).

## 4. Discussion

The greatest risk of death from pneumonia in childhood occurs during the neonatal period. GBS disease is typically marked by respiratory symptoms within the first few hours of life, reflecting an initial pulmonary focus of infection [37]. More than 80% of infected newborns present with respiratory pathology, which can progress to severe complications such as pneumonia [3]. Developmental differences in the immune system likely contribute to neonatal susceptibility compared to adults [38]. However, a lack of in-depth understanding of neonatal immunity in response to bacterial pneumonia limits the effectiveness of prevention and treatment strategies.

Traditionally, neutrophils have been considered the main culprits of inefficient bacterial clearance in neonates. Here, using a vertical transmission model of neonatal GBS pneumonia, we demonstrate that neutrophils are efficiently recruited into the lungs, but their phenotype varies with disease severity. We identified a subset of neutrophils expressing high levels of SiglecF present in neonates with moderate disease but absent in those with severe disease. The neonatal immune system is widely considered immature, which is commonly linked to an increased risk of pneumonia. Numerous studies have demonstrated impaired neutrophil amplification, mobilisation, and function, especially during severe neonatal sepsis, a condition that can follow or co-occur with pneumonia. This impaired response often leads to inadequate bacterial clearance, allowing the pathogen to spread and exacerbating disease severity [39,40,41]. To date, therapies aimed at enhancing granulopoiesis during sepsis, including the use of myeloid-specific growth factors, such as granulocyte-colony stimulating factor (G-CSF) and granulocyte/macrophage colony-stimulating factor (GM-CSF), have not proven effective in improving the survival of septic infants [42]. Elevated endogenous G-CSF levels in septic neonates suggest that end-organ unresponsiveness may contribute to this lack of therapeutic efficacy [43]. In this study, we found that neutrophils were efficiently recruited into the lungs of pups with neonatal pneumonia. This contrasts with our previous studies, which found that inhibiting TLR2 or IL-10 signalling improved neonatal survival by promoting effective neutrophil migration into infected tissues and enhancing bacterial clearance [44,45]. A possible explanation for these discrepancies is the infection route used. In the present study, we used a vertical transmission model of GBS, which more closely mimics human pathophysiology, rather than hematogenous bacterial dissemination. This reinforces the importance of using appropriate animal models that could better mimic human disease conditions. Our observation suggests that not the presence of neutrophils per se, but their phenotype, is a key determinant of bacterial clearance and disease progression. Importantly, mild histopathological changes were observed in both infected and uninfected pups at P1. These findings are likely reflecting normal postnatal lung adaptation processes such as alveolar expansion, fluid clearance, and transient immune cell infiltration, rather than infection-related pathology [46,47].

SiglecF-expressing neutrophils were only recently described and have been implicated in various diseases, including lung tumours [14,48], infarcted heart [49,50], airway inflammation and allergy [51], chronic kidney disease [52], pulmonary fibrosis [16], and bacterial infection [53]. These cells exhibit enhanced effector functions, including production of reactive oxygen species (ROS) and NETs formation [15,48,53]. Consistent with these reports, SiglecF^hi^ neutrophils isolated from the neonatal lungs exhibited higher phagocytic capacity, supporting their role in bacterial clearance and neonatal survival. Furthermore, in a less severe model of neonatal disease, where pathology was reduced and survival increased, neutrophils expressed SiglecF, supporting our hypothesis that increased severity may be linked to bacterial immune evasion through neutrophil inhibition.

In recent years, the role of neuropeptides in immune modulation has gained considerable attention, highlighting a unique crosstalk between neurons and immune cells. Peripheral neurons, including sensory neurons, can release neuropeptides locally, which in turn regulate immune responses via receptors expressed on immune cells [54,55]. CGRP, a potent vasodilator and neuropeptide primarily released by sensory neurons, has been shown to modulate immune responses [18,19,20,56,57]. Emerging studies have identified the CGRP pathway as a key factor in immune evasion strategies used by various pathogens, including GBS [6,21,22]. In an adult mouse model of GBS meningitis, genetic ablation of Nav1.8 sensory neurons decreased meningeal and brain colonisation [22]. This study found that GBS activated nociceptors to release CGRP in the meninges, contributing to meningitis pathogenesis, with systemic CGRP injection increasing CNS invasion [22]. Similarly, during *Streptococcus pyogenes* skin infection, CGRP release inhibited neutrophil recruitment to infected skin, causing dermonecrosis and weight loss [21]. The selective genetic ablation of TRPV1+ sensory neurons inhibited CGRP release, restoring neutrophil recruitment to infection sites [21]. In *Staphylococcus aureus* pneumonia, activation of lung nociceptors suppressed neutrophil infiltration [6]. In our model, although neutrophils were efficiently recruited to the lungs, their transition into a SiglecF^hi^ phenotype was impaired in severe disease, concomitant with increased expression of CGRP. While our data suggest a direct suppressive effect of CGRP on neutrophil activation, we acknowledge that this remains a hypothesis. It is also possible that CGRP upregulation acts as a regulatory mechanism to prevent excessive inflammation. CGRP has been shown to mediate immune suppression in pulmonary tissue, mainly by inhibiting neutrophil recruitment and γδ T cell-mediated defence in bacterial lung infections [6]. Additionally, CGRP can induce IL-10 [58], and systemic IL-10 levels increase during neonatal GBS infection [44,45]. Although we did not detect significant differences in IL-10 expression between moderate and severe groups, it remains possible that CGRP-mediated IL-10 induction occurs in a timing-dependent manner, or that CGRP contributes to immunosuppression through other regulatory pathways in this context. Thus, immunosuppression could diminish effective and protective antibacterial immunity, thereby increasing susceptibility, as previously described in a mouse model of neonatal GBS sepsis [44].

CGRP may also act indirectly by regulating γδ T cells, which we found to be a major source of IL-17A in the neonatal lungs. IL-17-producing γδ T cells are known to promote neutrophil recruitment during infections with *Mycobacterium tuberculosis*, *Escherichia coli*, and *Bordetella pertussis* [53,59,60]. In our study, their early activation supports a protective role in GBS pneumonia. IL-17 production upon nasal infection with *B. pertussis* is associated with the recruitment of SiglecF^+^ neutrophils, which are also crucial for protection [53,61]. Nevertheless, γδ T cells can also exacerbate pathology, as shown in cancer metastasis and inflammatory disorders, where the IL-17–neutrophil axis promotes disease progression [62,63]. Thus, whether γδ T cells in neonatal pneumonia predominantly contribute to protection or pathology requires further investigation.

While our study provides novel insights into the neuroimmune regulation of neonatal pneumonia, some limitations should be acknowledged. First, although we demonstrate an association between CGRP upregulation and impaired SiglecF^hi^ neutrophil development, we did not perform in vivo functional blockade of CGRP to confirm causality. Second, as other myeloid subsets can also express SiglecF, future work combining additional markers and single-cell approaches will be important to refine the identity of SiglecF^+^ neutrophils in this context. Finally, extrapolation from a murine vertical transmission model to human neonates requires caution, and clinical validation will be essential. Future studies should therefore investigate whether targeting the CGRP pathway could restore neutrophil function and improve outcomes in neonatal pneumonia.

In summary, our findings identify a protective SiglecF^hi^ neutrophil subset that supports bacterial clearance in neonatal GBS pneumonia. However, their development appears impaired in severe disease through CGRP-associated mechanisms. These results suggest that modulation of CGRP signalling may represent a promising avenue for restoring neutrophil function and improving outcomes in severe neonatal infections.

## Figures and Tables

**Table 1:** Histological score established for the evaluation of pulmonary alterations.

Score	Description
0	Absence of histological changes
1	Parenchymal alterations in 1 to 25% of the tissue examined
2	Parenchymal alterations in 26 to 50% of the tissue examined
3	Parenchymal alterations in 51 to 75% of the tissue examined
4	Parenchymal alterations in 76 to 100% of the tissue examined

**Table 2:** List of oligonucleotide sequences used.

Primer ID	Oligonucleotide Sequence (5′ to 3′)
*Tnfa* sense	GTGGAACTGGCAGAAGAG
*Tnfa* anti-sense	ATGAGAAGAGGCTGAGACA
*Il1b* sense	CAACCAACAAGTGATATTCTCCATG
*Il1b* anti-sense	GATCCACACTCTCCAGCTGCA
*Il17a* sense	CTCAGACTACCTCAACCGTTCCA
*Il17a* anti-sense	TTCCCTCCGCATTGACACA
*Il33* sense	TCCAACTCCAAGATTTCCCCG
*Il33* anti-sense	CATGCAGTAGACATGGCAGAA
*Il10* sense	ATTTGAATTCCCTGGGTGAGAAG
*Il10* anti-sense	CACAGGGGAGAAATCGATGACA
*Cgrp* sense	CCTGCAACACTGCCACCTGCG
*Cgrp* anti-sense	GAAGGCTTCAGAGCCCACATTG
*Hprt1* sense	TCAGTCAACGGGGGACATAAA
*Hprt1* anti-sense	GGGGCTGTACTGCTTAACCAG

## Data Availability

The original contributions presented in this study are included in the article material. Further inquiries can be directed to the corresponding author. Some of the data included in this manuscript are part of a Master’s dissertation (Ana Sofia Teixeira) and a PhD thesis (Inês Lorga) that are currently under institutional embargo and not publicly accessible. These theses have not been published in any peer-reviewed journal, conference, or public repository. Therefore, this does not constitute prior or dual publication.

## Supplementary Materials

The following supporting information can be downloaded at: https://www.mdpi.com/article/10.3390/microorganisms13092119/s1. Figure S1. Histopathology of the neonatal lung upon GBS pneumonia; Figure S2. Myeloid and γδ T cell profile after recovery from neonatal pneumonia; Figure S3. SiglecF^hi^ neutrophils are a distinct myeloid population; Figure S4. SiglecF^hi^ neutrophils do not differentiate in the spleen.
