# Supplementary material for: CGRP Suppresses Protective SiglecFhi Neutrophil Development in Neonatal Group B Streptococcus Pneumonia
Source: Microorganisms. 2025 Sep 11;13(9):2119. doi: 10.3390/microorganisms13092119 (PMC12473038; doi:10.3390/microorganisms13092119)
Supplement: Supplementary file 1 [file microorganisms-13-02119-s001.zip › Supplementary.pdf]

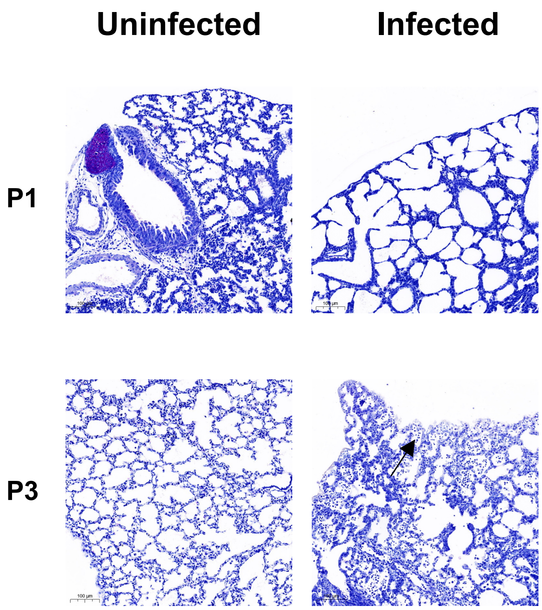

Figure S1. Histopathology of the neonatal lung upon GBS pneumonia. Pregnant C57BL/6 female mice were intravaginally inoculated with 4×10^4 CFU of GBS hyper virulent strain BM110, at gestational days 16th and 17th. Representative images of lung stained with Giemsa Hare showed for each time point. Scale bar, 100µm. Black arrow indicates neutrophils, in the alveoli.

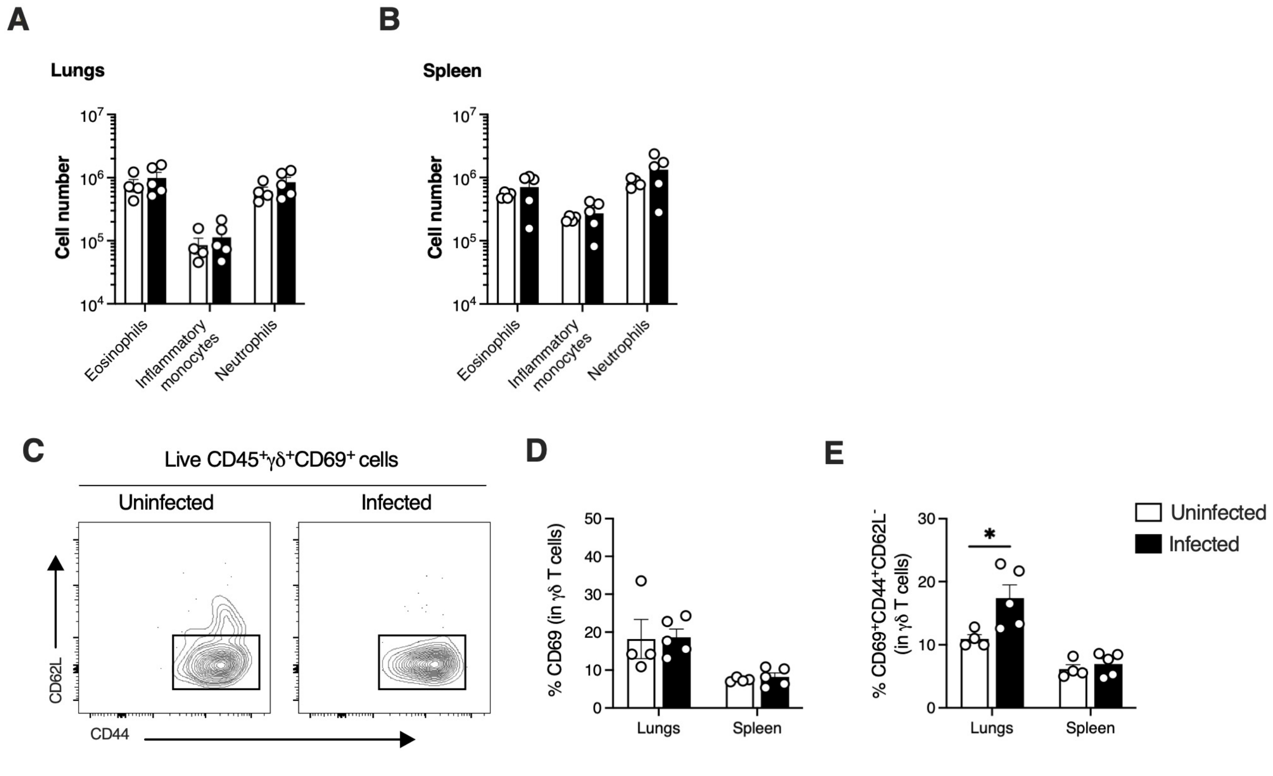

Figure S2. Myeloid and γδ T cell profile after recovery from neonatal pneumonia. Pregnant C57BL/6 female mice were intravaginally inoculated with 4 × 10^4 CFU of GBS hyper virulent strain BM110, at gestational days 16th and 17th. Animals from uninfected and infected groups were sacrificed at P15. Flow cytometry analysis of indicated myeloid cells in the lungs **(A)** and spleen **(B)**. Data is presented as mean ± SEM [n= 4 (uninfected); n= 5 (infected)]. Comparisons by Student's *t*-test. **(C)** Representative contour plots of tissue resident γδ T cell. The profile of tissue resident of γδ T cells was defined as $CD45^+γδ^+CD69^+CD44^+CD62L^-$. **(D-E)** Frequency of indicated cell populations among γδ T cells. Data is presented as mean ± SEM [n = 4 (uninfected); n = 5 (infected)]. Comparisons by Student's *t*-test. Each symbol indicates data from single pup. Results are representative of two independent experiments. Statistical differences (P values) between groups are indicated. * $P < 0.05$.

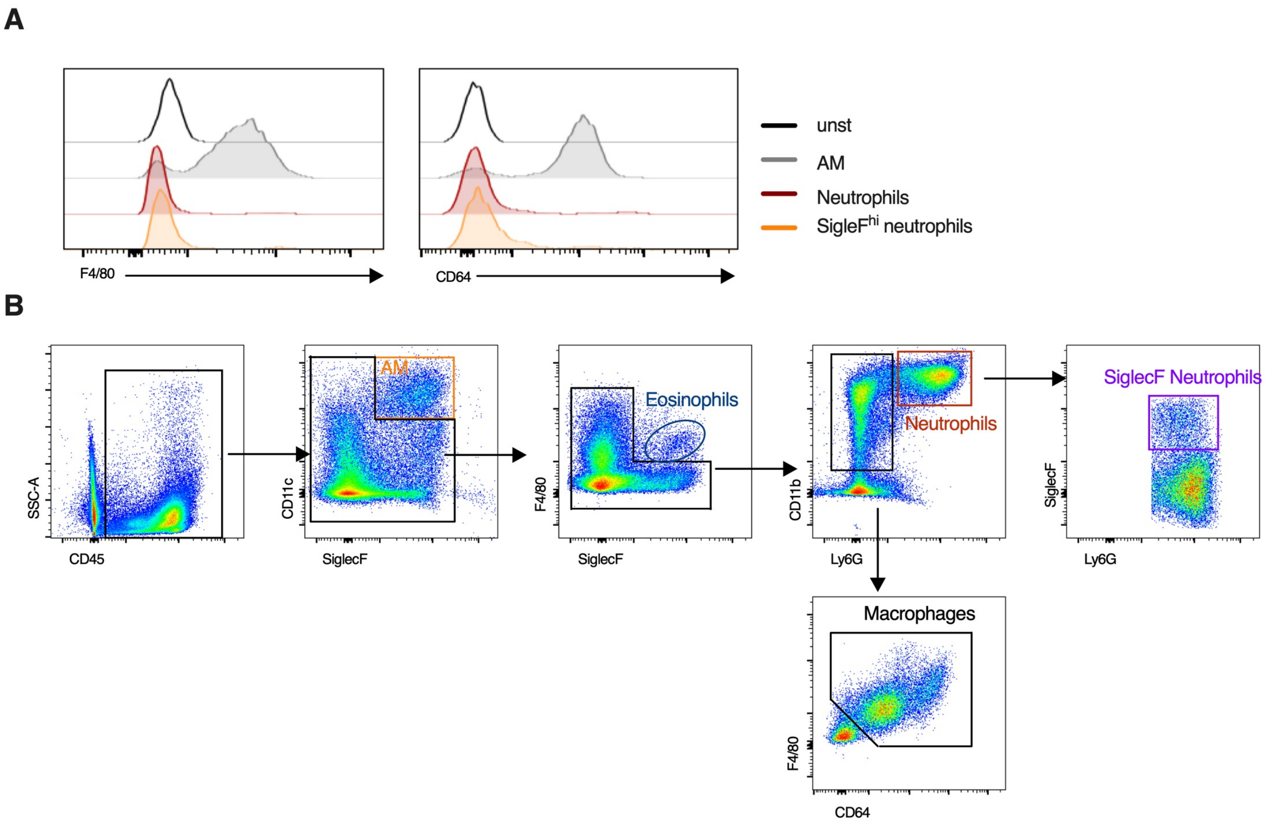

Figure S3. SiglecF^{hi} neutrophils are a distinct myeloid population. Pregnant C57BL/6 female mice were intravaginally inoculated with 4 × 10^{4} CFU of GBS hyper virulent strain BM110, at gestational days 16th and 17th. Animals were sacrificed at P3. **(A)** The expression of F4/80 and CD64, along with their mean fluorescence intensity (MFI), in lung immune cells of infected mice **(B)**. Representative gating strategy used to define myeloid cells, gated within live CD45^{+} cells.

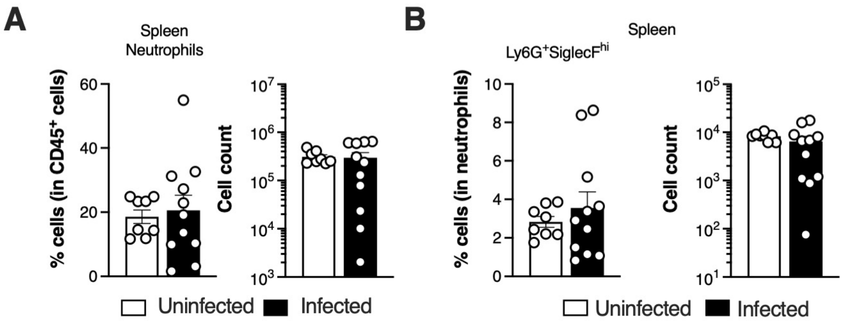

Figure S4. SiglecF^{hi} neutrophils do not differentiate in the spleen. Pregnant C57BL/6 female mice were intravaginally inoculated with 4 × 10^4 CFU of GBS hyper virulent strain BM110, at gestational days 16th and 17th. Animals were sacrificed at P3. **(A)** Frequency and number of neutrophils in the spleen. Data is presented as mean ± SEM [n = 8 (uninfected); n = 11 (infected)]. Each symbol represents data from single pup. Comparisons by Student's t test. **(B)** Frequency and number of SiglecF^{hi} neutrophils in the spleen. Data is presented as mean ± SEM [n = 8 (uninfected); n = 11 (infected)]. Each symbol represents data from single pup. Comparisons by Student's t-test. Results are pooled from two to four independent experiments. Statistical differences (P values) between groups are indicated.
